# The Effects of Open and Closed Suction Methods on Occurrence of Ventilator Associated Pneumonia; a Comparative Study

**Published:** 2020-01-11

**Authors:** Seyed Hossein Ardehali, Alireza Fatemi, Seyedeh Fariba Rezaei, Mohammad Mehdi Forouzanfar, Zahra Zolghadr

**Affiliations:** 1 Department of Anesthesiology & Critical Care, Shohadaye Tajrish Hospital, Shahid Beheshti University of Medical Sciences, Tehran, Iran.; 2 Men's Health and Reproductive Health Research Center, Shahid Beheshti University of Medical Sciences, Tehran, Iran.; 3 Emergency department, Shohadaye Tajrish Hospital, Shahid Beheshti University of Medical Sciences, Tehran, Iran.; 4 Department of Biostatistics, School of Allied Medical Sciences, Shahid Beheshti University of Medical Sciences, Tehran, Iran.

**Keywords:** Pneumonia, Ventilator-Associated, Respiration, Artificial, critical care, intensive care units, suction

## Abstract

**Introduction:**

Endotracheal suctioning is a method commonly used to clean airway secretions in patients under mechanical ventilation (MV). This study aimed to compare the effects of open and closed suction methods on the occurrence of ventilator associated pneumonia (VAP).

**Methods:**

This comparative study was carried out on adult intensive care unit (ICU) patients in need of MV for more than 48 hours, from October 2018 to January 2019. Patients were randomly allocated to either closed tracheal suction system (CTSS) group or open tracheal suction system (OTSS) group. Patients were monitored for developing VAP within 72 hours of intubation and the findings were compared between groups.

**Results:**

120 cases with the mean age of 57.91±19.9 years were randomly divided into two groups (56.7% male). The two groups were similar regarding age (p = 0.492) and sex (p = 0.713) distribution. 22 (18.3%) cases developed VAP (12 (20%) in OSST group and 10 (16.7%) in CSST; p = 0.637). The most prevalent bacterial causes of VAP were Acinetobacter_Baumannii (72.7%), Klebsiella pneumoniae (18.2%), and Methicillin-Resistant Staphylococcus aureus (9.1%), respectively. There was not any significant difference between groups regarding the mean duration of remaining under MV (p = 0.623), mean duration of hospitalization (p = 0.219), frequency of VAP (p = 0.637), and mortality (p = 0.99).

**Conclusion:**

It seems that type of endotracheal suction system (OSST vs. CSST) had no effect on occurrence of VAP and other outcomes such as duration of need for MV and ICU stay as well as mortality.

## Introduction:

Ventilator-associated pneumonia (VAP) is one of the most common nosocomial infections in intensive care units (ICUs), and is associated with a high rate of morbidity and high cost of care (1). Therefore, any intervention to reduce VAP will result in reducing costs, morbidity and mortality (2). Endotracheal tube (ETT) suctioning is an essential procedure in patients undergoing mechanical ventilation (MV) with intubation to keeping the airways open through removal of accumulated pulmonary secretions (3). In addition, performing suction accurately is important to prevent VAP (4).

Two different methods are used for ETT suctioning, open tracheal suction system (OTSS), and closed tracheal suction system (CTSS). OTSS method requires participation of two nurses and may lead to temporary disruption of ventilation and oxygen supply due to disconnection of the patient from ventilation device during suctioning and the most important risk factor in this method is hypoxia (5, 6). However, in CTSS method, ETT suctioning can be administrated through connections in closed suction set and while the ventilation is being performed without disconnecting the patient from the ventilator (7). The effect of open and closed suction methods on preventing VAP is still an open field to be explored, because the results of previous studies are contradictory (8-12). Some studies showed that there was no difference between closed and open endotracheal suction systems in terms of VAP development (9, 11, 12). On the other hand, Alipour et al. (10) and Fakhar et al. (13) showed that closed suction is associated with a lower risk of VAP compared to open suction. Therefore, we conducted this comparative study to compare the effects of open and closed suction methods on VAP in mechanically ventilated patients.

## Methods:

***Study design and setting***

This comparative study was conducted prospectively on ICU-admitted patients in Shohadaye Tajrish Hospital, Tehran, Iran, from October 2018 to January 2019. Patients were randomly divided into two groups of CTSS and OTSS and the outcomes (VAP occurrence, mortality, ICU stay, and duration of need to MV) of the two groups were compared. This study was performed after receiving approval from the ethics committee of Shahid Beheshti University of medical sciences (Ethics code: IR.SBMU.MSP.REC.1398.69). A written consent was obtained from patients or legal guardians of patients.

***Participants***

Adult patients who underwent MV for more than 48 hours were included in the study. Patients unwilling to participate or those with pneumonia or any other underlying respiratory diseases that increase the risk of pneumonia at the time of admission and patients who had remained intubated for more than 48 hours before admission were excluded from the study. 

***Data gathering***

Demographic characteristics of patients (age and sex), duration of MV and the length of hospital stay as well as developing VAP within 72 hours of intubation were examined and recorded by an intensivist for all cases.

**Appendix 1:** Clinical Pulmonary Infection Score used for diagnosis of ventilator-associated pneumonia

**Criterion**	**Score**
**Temperature, °C**	
≥36.5 and ≤38.4	0
38.5 and ≤38.9	1
≥39 and ≤ 36	2
**Leukocyte count (1/mm** ^3^ **)**	
≥4,000 and ≤11,000	0
<4,000 or >11,000	1
+band forms ≥500	2
**Oxygenation, PaO**_2_ **/FiO**_2_ ** (mmHg)**	
>240 or ARDS	0
≤240 and no evidence of ARDS	2
**Pulmonary radiography**	
No infiltrate	0
Diffused (or patchy) infiltrate	1
Localized infiltrate	2
**Tracheal secretions**	
<14+	0
≥14+	1
+ purulent secretion	2
Culture of tracheal aspirate*	
Pathogenic bacteria cultured ≤1+ or no growth	0
Pathogenic bacteria cultured >1+	1
+ same pathogenic bacteria seen on the Gram stain >1+	2

*: Semi-quantitative: 0-1-2 or 3+. ARDS: Acute respiratory distress syndrome.

**Table 1:** Comparing the outcomes between open tracheal suction system (OTSS) and closed tracheal suction system (CTSS) groups

**Outcomes**	**Type of suction**		**p**
	**OTSS (n=60)**	**CTSS (n=60)**	
**Ventilator associated pneumonia**			
Yes	12 (20.0)	10 (16.7)	0.637
No	48 (80.0)	50 (83.3)	
**Duration of ventilation**			
Mean ± SD	13.47±10.83	12.62±7.82	0.623
Range (days)	5-65	5-47	
**Hospitalization days**			
Mean ± SD	21.83±12.72	19.20±10.50	0.219
Range (days)	8-73	8-55	
**Microorganism**			
Acinetobacter_Baumannii	9 (56.2)	7 (43.8)	0.96
Klebsiella pneumoniae	2 (50.0)	2 (50.0)	
MRSA	1 (50.0)	1 (50.0)	
No	48 (49.0)	50 (51.0)	
**Death**			
Yes	39 (65.0)	39 (65.0)	0.99
No	21 (35.0)	21 (35.0)	

Data are presented as mean ± standard deviation (SD) or frequency (%). MRSA: Methicillin-resistant Staphylococcus aureus.

***Procedure***

Patients who met the inclusion criteria were randomly divided into two groups of CTSS and OTSS based on endotracheal suction methods using sequential randomization. In both groups, throat samples from endotracheal tubes and ventilator tubing were taken to determine the rate of colonization. Conventional bacteriological methods were used for identification of isolated micro-organisms. Antimicrobial susceptibility test was performed using Disk Diffusion method according to CLSI (Clinical and Laboratory Standards Institute) guidelines (14). 

Suction methods were administrated based on the protocol of American Association for Respiratory Care (AARC) (15). Endotracheal suction was performed by experienced ICU nurses. In the OTSS group, suctioning was performed using single use catheters with full barrier measures (hand washing and use of gloves). Patients were pre-oxygenated for 2 minutes before suctioning. In the CTSS group, the system used for respiratory system suctioning was (Vital-Cath TM 72 Closed Suction Systems) and suction catheter was changed every 48 hours. Similar to the other group, patients were pre-oxygenated, and suctioning was performed without disconnection from the ventilator. The following VAP prophylaxis strategies were used in all patients: head elevation (30-40°), heat and moisture exchanger (HME) for humidification, protocolized sedation and enteral nutrition, performing suction only when necessary, avoiding routine change of the respiratory circuit unless necessary, mouth washing with chlorhexidine in each shift, pantoprazole for prophylaxis of stress ulcer, verification of gastric residual volume in each shift, avoidance of unnecessary extubation or intubation, maintenance of cuff pressure between 20-30 mmHg and continuous aspiration of subglottic secretions. All interventions were done by one medical doctor and two nurses who provided care for both groups.

Diagnosis of VAP was performed based on clinical pulmonary infection score (CPIS) (16). Patients were monitored for 72 hours from suctioning and examined by an infectious disease specialist. Bacterial pneumonia index calculated based on persistent infiltration in the chest X-ray, body temperature, white blood cell count, airway discharges, ratio of arterial blood oxygen to inhaled oxygen, and culture and smear of lung discharges were recorded. Patients were considered to have pneumonia if they received a score higher than 6 (appendix 1) (17).

***Statistical analysis***

Qualitative variables were reported as percentage and quantitative variables as mean± standard deviation. We used Student t test and chi-square test for detection of differences between the two groups. Fisher’s exact test was used for qualitative analysis when necessary. For analyzing data, SPSS version 21 (SPSS Inc., IMB Corporation, Chicago, Illinois, USA) was used. P value equal to or less than 0.05 was considered statistically significant.

## Results:

120 cases with the mean age of 57.91±19.9 (17 – 94) years were randomly divided into two groups of OSST or CSST (56.7% male) with equal participants. The two groups were similar regarding age (p = 0.492) and sex (p = 0.713) distribution. 22 (18.3%) cases developed VAP (12 (20%) in OSST group and 10 (16.7%) in CSST; p = 0.637). The most prevalent bacterial causes of VAP were Acinetobacter_Baumannii (72.7%), Klebsiella pneumoniae (18.2%), and Methicillin-Resistant Staphylococcus aureus (9.1%), respectively. No statistically significant difference was detected between groups regarding the frequency of bacterial causes VAP (p = 1.000). Table 1 compares the outcomes of patients between groups. There was not any significant difference between groups regarding the mean duration of undergoing MV (p = 0.623), mean duration of hospitalization (p = 0.219), frequency of VAP (p = 0.637), and mortality (p = 0.99). 

## Discussion:

The results revealed that the type of endotracheal suction system (OSST vs. CSST) had no effect on development of VAP and ICU outcome. In addition, our results did not show any significant difference between the two groups regarding length of ICU stay and duration of MV and mortality rate, which is similar to the results of Combes et al. (18), Topeli et al. (12), Ozcan et al. (19) and Hamishekar et al. (9).

Different studies that assessed the effect of open and closed suction on incidence of VAP showed controversial findings (9, 10, 12). Our finding is consistent with some previous studies that showed no statistically significant difference between OSST and CSST endotracheal suctioning systems in terms of VAP development (20, 21). In a systematic review by Subirana et al., 16 clinical trials were assessed; their results showed that using open or closed suction methods had no effect on VAP (22). A prospective randomized study, which was carried out on 100 patients in surgical ICU by Hamishekar et al. to evaluate the effect of CTSS versus OTSS did not show any statistically significant effect on VAP incidence in multivariate analysis (9). However, in contrast to our findings, some studies showed that closed suction method has superiority over open method in reducing the incidence of VAP (10, 13). In a prospective clinical trial performed by David et al. clinical results of OSST and CSST were assessed in 200 patients under MV in India; they found that using closed suction reduced the incidence of VAP. However, mortality rate and hospital stay in ICU were the same in both groups (23). The controversial/contradicting results in different studies can have many reasons such as small sample size, inappropriate inclusion or exclusion criteria such as including patients with underlying respiratory diseases, short duration of study period, not teaching the principles of using closed suction to nurses, and not using the VAP prophylaxis strategies in open suction method. 

In the present study, we tried to solve the above-mentioned limitations by using appropriate inclusion and exclusion criteria, including appropriate number of patients in both groups, properly training nurses for using closed suction, and using VAP prophylaxis strategies as a health principle. Considering that both endotracheal suction systems have advantages and disadvantages, it seems that the incidence of VAP can be reduced by using aseptic precautions based on signs and symptoms, as well as the correct use of guidelines in both suction systems. 

The distribution of micro-organisms causing VAP was different in various studies, which could be due to differences in patient demographics, methods of diagnosis, duration of hospitalization, ICU stays, and antibiotic policy (24, 25). In the present study, Acinetobacter_Baumannii was the most common isolated pathogen (72.7%) in patients with VAP followed by Klebsiella pneumoniae (18.2%) and methicillin-resistant Staphylococcus aureus (MRSA) (9.1%). Consistent with the present investigation, a study by Dey et al. reported Acinetobacter species (48.94%) as the most common isolate from early-onset and late-onset VAP (26). Previous studies have shown that Acinetobacter species ranked fifth among the causative organisms of VAP (27-29). Bozorgmehr et al. reported that acinetobacter baumannii and klebsiella pneumoniae were the most common germs growing in sputum cultures and most of them were pan drug resistance (PDR) or extensive drug resistance (XDR) (30).

Our study limitation is that type of VAP (early or delayed) was not studied, which is suggested to be further evaluated in future studies. In addition, data regarding underlying diseases and cause of intubation were not recorded; however, they may play a role in increasing mortality due to ventilation.

In conclusion, according to the findings of this study, the use of CSST has no superiority over OSST in reducing VAP incidence of and it has no effect on ICU outcome. The incidence of VAP was remarkable in both groups, which led to an increase in hospitalization and mechanical ventilation in these patients. In addition, Acinetobacter_Baumannii was found to be the most common isolated pathogen followed by K. pneumoniae and MRSA. 

## Conclusion:

It seems that type of endotracheal suctioning system (OSST vs. CSST) had no effect on occurrence of VAP and other outcomes such as duration of MV and ICU stay as well as mortality.
